# Real‐world clinical outcomes of the combination of anti‐PD‐1 antibody, trastuzumab, and chemotherapy for HER2‐positive gastric/gastroesophageal junction cancer

**DOI:** 10.1002/cam4.5722

**Published:** 2023-03-13

**Authors:** Ju Yang, Zhan Shi, Xin Zhang, Qin Liu, Xiaobin Cui, Lin Li, Baorui Liu, Jia Wei

**Affiliations:** ^1^ The Comprehensive Cancer Centre of Drum Tower Hospital Medical School of Nanjing University and Clinical Cancer Institute of Nanjing University Nanjing China; ^2^ The Department of Pathology of Drum Tower Hospital Medical School of Nanjing University Nanjing China

**Keywords:** anti‐PD‐1 antibody, chemotherapy, gastric/gastroesophageal junction cancer, HER2, trastuzumab

## Abstract

**Background:**

Previous clinical trials indicated the addition of anti‐PD‐1 antibody remarkably improved the efficacy of trastuzumab and chemotherapy in patients with HER2‐positive gastric/gastroesophageal junction (GEJ) cancer. However, no real‐world experiences have been reported yet.

**Methods:**

We retrospectively analyzed 1212 patients with gastric/GEJ cancer treated at Nanjing Drum Tower Hospital between 2019 and 2022. Among 138 patients with HER2‐positive gastric/GEJ cancer, 47 patients receiving at least two doses of the combination regimen with anti‐PD‐1 antibody, trastuzumab, and chemotherapy were recruited in the study population, and 38 out of 47 patients with measurable disease were included in the efficacy population. Progression‐free survival (PFS), objective response rate (ORR), disease control rate (DCR), and toxicity profiles were reported.

**Results:**

In the study population, 37 (78.7%) received the study therapy as a first‐line treatment. In the efficacy population, the ORR and DCR were 76.3% and 94.7%, respectively. The overall median PFS was 9.1 months (95% confidence interval [CI] 6.3–11.9 months). For the first‐line treatment, the mPFS was 10 months, and 7 months for the second‐line. Among 14 patients who failed the study treatment, three (21.4%) developed brain metastasis as the first failure site. No significant association was found between PFS and the expression of PD‐L1. 22.2% of patients developed grade 3 treatment‐related adverse events (TRAEs). No treatment‐related grade ≥4 adverse events or deaths occurred.

**Conclusion:**

This real‐world study validated the combination regimen's high efficacy and good tolerance in patients with HER2‐positive gastric/GEJ cancer. An increased incidence of brain metastasis was observed in patients who failed this regimen.

## 1 INTRODUCTION

Gastric cancer (GC) is the fifth most commonly diagnosed cancer and the fourth leading cause of cancer‐related death worldwide and ranks second for incidence and third for mortality in China.^1^ Currently, the overall 5‐year survival rate is only 35.1% in China.^2^ Over 80% of GC patients are diagnosed at an advanced stage in China^3^ and with a median overall survival (OS) of 10–14 months.^4^

HER2 is overexpressed or amplified in about 20% of patients with GC.^5^ In the Chinese population, the positive rate is 10.8%–12.5%.^6^, ^7^ HER2 overexpression/amplification is solidly associated with a more aggressive disease in patients with GC.^8^ HER2 positivity defined as HER2 immunohistochemistry (IHC) scores of 3+ or 2+ with fluorescent in situ hybridization‐positive (FISH+). The phase III ToGA trial established the addition of trastuzumab (a humanized monoclonal antibody that targets HER2) to chemotherapy in advanced HER2‐positive GC based on the improved median OS (16.0 vs. 11.8 months).^9^

With the development of immunotherapy in recent years, the addition of checkpoint inhibitors to HER2‐targeted therapy significantly improved outcomes in patients with advanced HER2‐positive GC. Two single‐arm, phase II studies (NCT02954536, NCT02901301) have demonstrated the clinical efficacy and safety for the combination of anti‐PD1 antibody, trastuzumab, and chemotherapy in metastatic HER2‐positive GC patients.^10^, ^11^, ^12^ To further investigate the combination, Janjigian et al. conducted a randomized, double‐blind, placebo‐controlled phase III KEYNOTE‐811 trial. In the protocol‐specified first interim analysis, the objective response rate (ORR) was significantly improved in the pembrolizumab group compared with the placebo group (74.4% vs. 51.9%) (NCT03615326).^13^

Though impressive results were achieved in metastatic HER2‐positive GC patients treated by the combination of pembrolizumab with chemotherapy/trastuzumab, to our knowledge, no real‐world experiences have been reported yet. To address this gap, we retrospectively analyzed the metastatic HER2‐positive GC patients treated by the combination of checkpoint inhibitor, chemotherapy, and trastuzumab between 2019 and 2022 in our institution in a real‐world setting.

## 2 METHODS

### 2.1 Patient eligibility

This study was performed under the guidelines of the Ethics Committee of Nanjing Drum Tower Hospital, which is an academic institution affiliated with Nanjing University. Patients treated in our hospital will be informed of the potential research use of personal information. Only patients who agreed with and signed the consent were included in our study. The eligibility criteria included: pathologically confirmed gastric/gastroesophageal junction (GEJ) cancer; at an advanced stage; HER2 IHC score 3+ or 2+ with FISH+; treated by the combination of immune checkpoint inhibitor, trastuzumab, and chemotherapy between 2019 and 2022. The exclusion criteria included: HER2‐negative; patients with HER2‐positive GC receiving adjuvant chemotherapy or neoadjuvant therapy.

### 2.2 Treatment

Trastuzumab was intravenously given (i.v.) with a loading dose of 8 mg/kg on Day 1 and a subsequent maintenance dose of 6 mg/kg every 3 weeks. Patients intravenously received a flat dose of pembrolizumab 200 mg or sintilimab 200 mg every 3 weeks. The chemotherapy regimen included platinum plus fluoropyrimidine. The first‐line chemotherapy was given as follows: intravenous oxaliplatin 130 mg/m^2^ on Day 1 and oral S‐1 40–60 mg twice daily, Days 1–14, every 3 weeks. If S‐1 was not tolerated, oral capecitabine 2–3 g was given twice daily, Days 1–14, every 3 weeks. The second‐line chemotherapy regimen included intravenous nab‐paclitaxel 100–120 mg/m^2^ on Days 1, 8 every 3 weeks, or intravenous docetaxel 75 mg/m^2^ on Day 1 every 3 weeks, or irinotecan 150 mg/m^2^ on Day 1 every 2 weeks. Dose modifications were indicated if untolerated toxicities occurred. The treatment was given until patients' refusal, disease progression, or unacceptable toxicities.

### 2.3 Molecular biomarkers

We retrospectively collected the information of molecular biomarkers, including HER2 status, mismatch repair (MMR) status, expressions of PD‐L1, and Epstein–Barr virus (EBV), from the department of pathology. HER2 IHC scores 3+ or 2+ with FISH‐positive were defined as HER2‐positive. MMR status was estimated by using an MMR IHC panel to detect expressions of the MMR proteins including MLH1, PMS2, MSH2, and MSH6. Expressions of the four proteins indicate an MMR‐proficient tumor, and loss of expressions suggests an MMR‐deficient (dMMR) tumor.^14^, ^15^ The PD‐L1 expression was detected by VENTANA PD‐L1 (SP142) assay. The combined positive score (CPS) was defined as the number of PD‐L1 positive cells including tumor, lymphocytes, and macrophages divided by total tumor cells. The FISH assay was used to determine the EBV status.

### 2.4 Response evaluation and follow‐up

Treatment response was assessed every 6–8 weeks with magnetic resonance imaging (MRI) or computed tomography according to response evaluation criteria in solid tumors (RECIST version 1.1).^16^ In this study, we investigated ORR, disease control rate (DCR), median progression‐free survival (PFS), and toxicities. The ORR is the proportion of patients who have a complete response (CR) or partial response (PR) to therapy, and DCR is ORR plus the proportion of patients with stable disease. Adverse events were evaluated during follow‐up and for 30 days after the last treatment. All treatment‐related toxicities were assessed according to the Common Toxicity Criteria for Adverse Events version 4.03.

### 2.5 Statistical analyses

We used the SPSS 20.0 version to analyze the data. Associations between patients' and treatment‐related factors and PFS were estimated with the Kaplan–Meier analysis and a Cox proportional hazards model. *p*‐values ≤0.05 were considered statistically significant.

## 3 RESULTS

### 3.1 Recruitment process of our study

**FIGURE 1:**
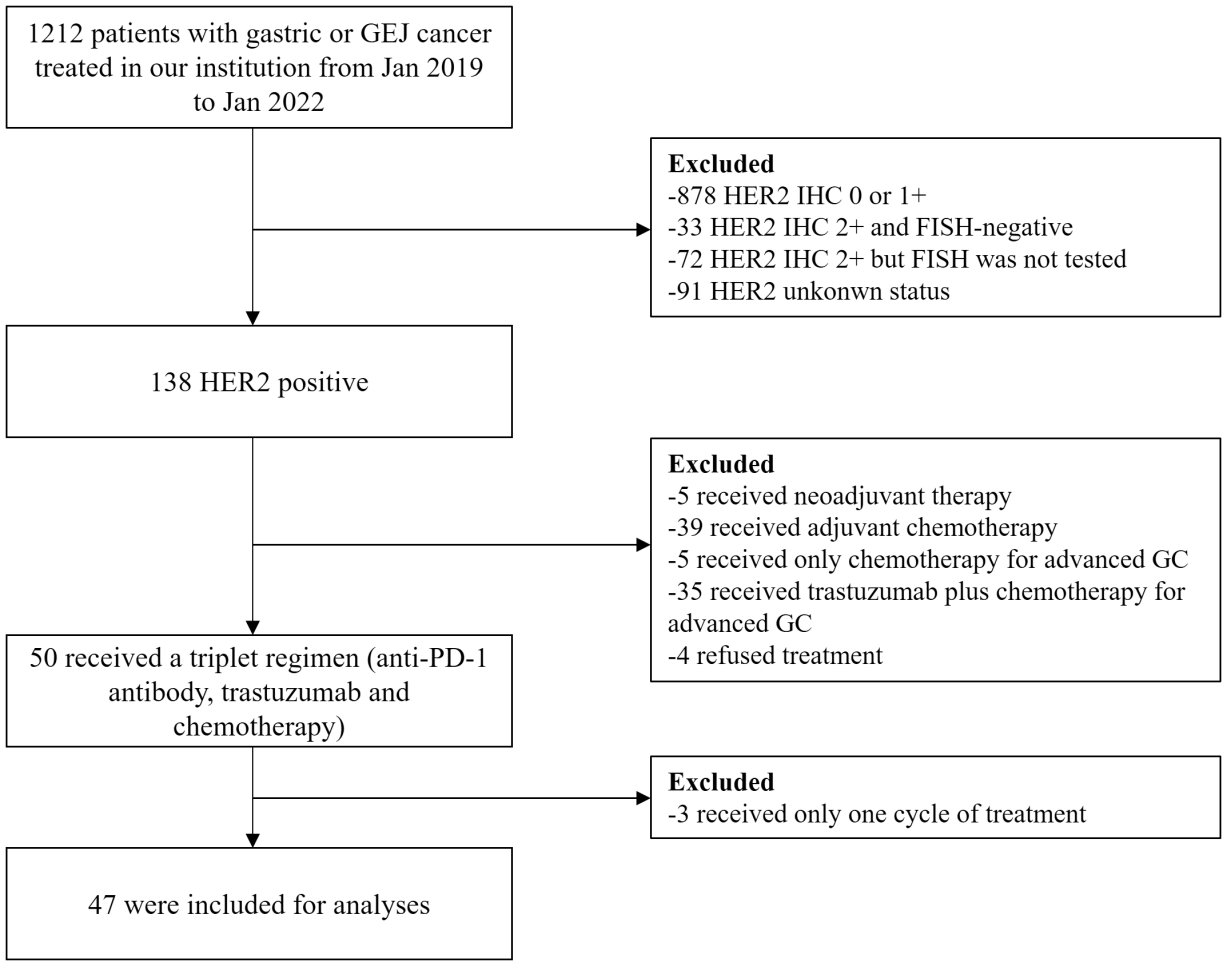
Flowchart of the recruitment process. FISH, fluorescence in situ hybridization; GC, gastric cancer; GEJ, gastroesophageal junction; HER2, human epidermal growth factor receptor 2; IHC, immunohistochemistry; PD‐1, programmed cell death 1.

The recruitment process of our study is shown in Figure 1. In total, 1212 patients were treated for gastric/GEJ cancer in our department from January 2019 to January 2022. There were 138 HER2‐positive patients (138/1212, 11.4%). Among HER2‐positive patients, 50 patients received a combination regimen with anti‐PD‐1 antibody, trastuzumab, and chemotherapy for advanced or metastatic disease. We excluded three patients who received only one dose of the study therapy and refused the subsequent treatment. Finally, 47 patients were recruited for analyses of treatment efficacy and adverse events.

### 3.2 Patients' characteristics

As shown in Table 1, the median age of the study population was 66 years (range 28–85 years), and most patients (80.9%) were male. In 42.6% of the recruited patients, the primary tumor location was at the GEJ. 78.7% of the patients received the combination regimen with anti‐PD‐1 antibody, trastuzumab, and chemotherapy as a first‐line treatment. Most patients (74.5%) had ≥2 metastatic sites. And the most common metastatic sites were lymph nodes (80.9%), liver (48.9%), bone (19.1%), and lung (17.0%). No patients were with dMMR status. One patient (2.1%) was EBV‐positive. In HER2‐positive patients, 76.6% of patients were with HER2 IHC 3+, and 23.4% of patients were HER2 IHC 2+ and FISH‐positive. Twenty‐five patients (53.2%) had tumors with CPS ≥1.

**TABLE 1:** Patients' characteristics.

	No. of patients (*N* = 47)	%
Age (years)		
Median	66 (range 28–85)	
<65	22	46.8
≥65	25	53.2
Sex		
Male	38	80.9
Female	9	19.1
ECOG PS		
0	10	21.3
1	37	78.7
Family history of cancer		
Yes	13	27.7
No	34	72.3
Primary tumor site		
Gastric	27	57.4
Gastroesophageal junction	20	42.6
Prior surgery		
Yes	25	53.2
No	22	46.8
Previous lines of chemotherapy		
None (first‐line)	37	78.7
One (second‐line)	10	21.3
Chemotherapy regimen		
Oxaliplatin	32	68.1
Capecitabine	5	10.6
S‐1	28	59.6
Taxanes	7	14.9
Irinotecan	2	4.3
Lauren classification		
Intestinal	15	31.9
Diffuse	3	6.4
Mixed	5	10.6
Unknown	24	51.1
Metastastic sites		
1	12	25.5
≥2	35	74.5
Metastatic sites		
Liver	23	48.9
Lung	8	17.0
Bone	9	19.1
Lymph nodes	38	80.9
Others	2	4.3
Molecular biomarkers		
MMR status		
pMMR	42	89.4
dMMR	0	0
Unknown	5	10.6
EBV status		
Positive	1	2.1
Negative	38	80.9
Unkown	8	17.0
HER2 status		
IHC 2+ and FISH‐positive	11	23.4
IHC 3+	36	76.6
PD‐L1 expression		
CPS<1	18	38.3
CPS≥1	25	53.2
Unknown	4	8.5

Abbreviations: CPS, combined positive score; dMMR, mismatch repair‐deficient; EBV, Epstein–Barr virus; ECOG PS, eastern cooperative oncology group performance status; FISH, fluorescence in situ hybridization; HER2, human epidermal growth factor receptor 2; IHC, immunohistochemistry; MMR, mismatch repair; PD‐L1, programmed cell death ligand 1; pMMR, mismatch repair‐proficient.

### 3.3 Treatment efficacy and clinical outcomes in the efficacy population

In the study population, the treatment responses of six patients have not been assessed until the last follow‐up. Two patients were given the anti‐PD‐1 antibody after the treatment failure of trastuzumab and were not included in the efficacy population. But the addition of immunotherapy did not control the disease progression in these two patients. One patient had a non‐measurable lesion (ascites) by RECIST 1.1 criteria and was excluded in the efficacy population either. Eventually, 38 patients with measurable disease according to RECIST 1.1 criteria were included in the efficacy population.

**FIGURE 2:**
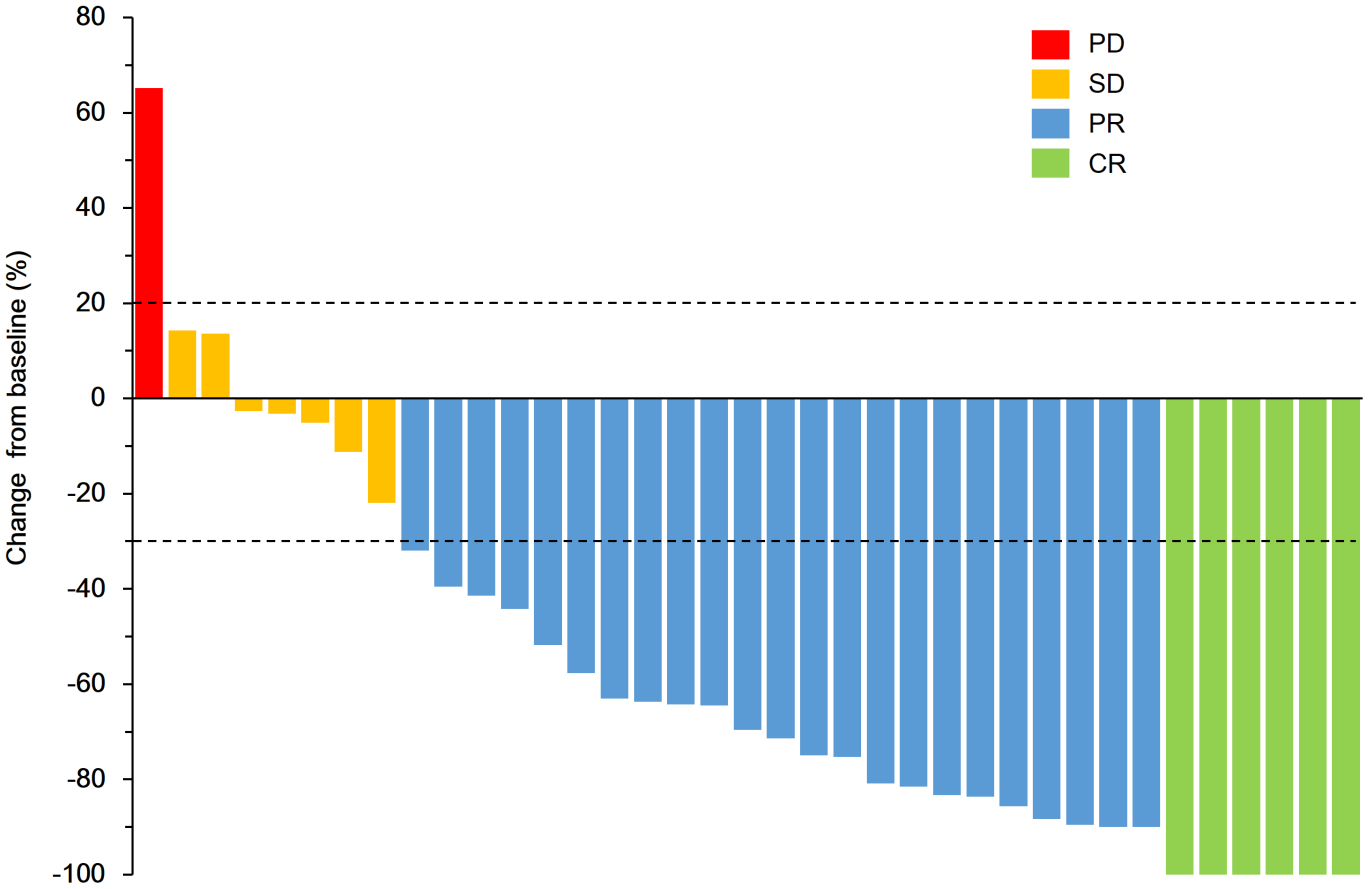
Best treatment responses. Only patients who had RECIST‐measurable disease at baseline and at least one post‐baseline evaluation were included (*N* = 36). CR, complete response; PD, progressive disease; PR, partial response; RECIST, response evaluation criteria in solid tumors; SD, stable disease.

In the efficacy population, there were 10 patients receiving the study therapy as a second‐line treatment (Table 2). These 10 patients developed disease progression during or within 6 months after the completion of adjuvant chemotherapy. Therefore, the study therapy was considered a second‐line treatment for these 10 patients. For all patients in the efficacy population, six patients (15.8%) achieved CR, and 23 patients (60.5%) achieved PR. The overall ORR was 76.3% (29/38), and the overall DCR was 94.7% (36/38). Best treatment responses are shown in Figure 2. The ORR for the first‐line and second‐line treatments was 82.1% and 60%, respectively. Until the latest follow‐up, 14 patients failed the study treatment. Eight patients were in the first‐line treatment group and six patients were in the second‐line treatment group. The most common first failure sites were the lymph nodes (5/14, 35.7%) and the liver (3/14, 21.4%). Interestingly, we found three patients developed brain metastasis as the first failure site (21.4%, 3/14) (Table 2).

**TABLE 2:** Treatment response and first treatment failure site in the efficacy population.

Best overall response	First‐line (*N* = 28) (%)	Second‐line (*N* = 10) (%)	Overall (*N* = 38) (%)
Complete response	4 (14.3)	2 (20.0)	6 (15.8)
Partial response	19 (67.9)	4 (40.0)	23 (60.5)
Stable disease	5 (17.9)	2 (20.0)	7 (18.4)
Progressive disease	0	2 (20.0)	2 (5.3)
Objective response	23 (82.1)	6 (60.0)	29 (76.3)
Disease control	28 (100.0)	8 (80.0)	36 (94.7)

First failure site	First‐line (*N* = 8) (%)	Second‐line (*N* = 6) (%)	All patients with treatment failure (*N* = 14) (%)
Liver	2 (25.0)	1 (16.7)	3 (21.4)
Lung	2 (25.0)	0	2 (14.3)
Bone	0	2 (33.3)	2 (14.3)
Lymph nodes	3 (37.5)	2 (33.3)	5 (35.7)
Brain	1 (12.5)	2 (33.3)	3 (21.4)
Others	1 (12.5)	1 (16.7)	2 (14.3)

**FIGURE 3:**
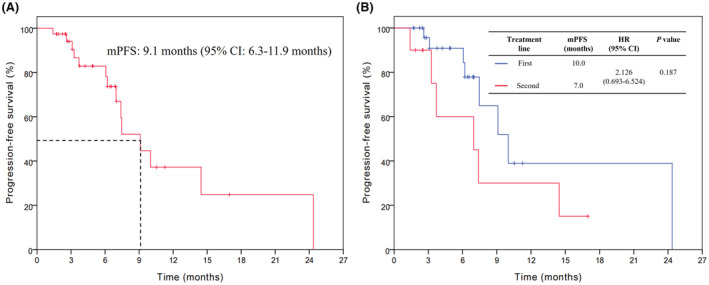
Kaplan–Meier analyses of progression‐free survival (A) and according to treatment line (B). CI, confidence interval; HR, hazard ratio; mPFS, median progression‐free survival.

**FIGURE 4:**
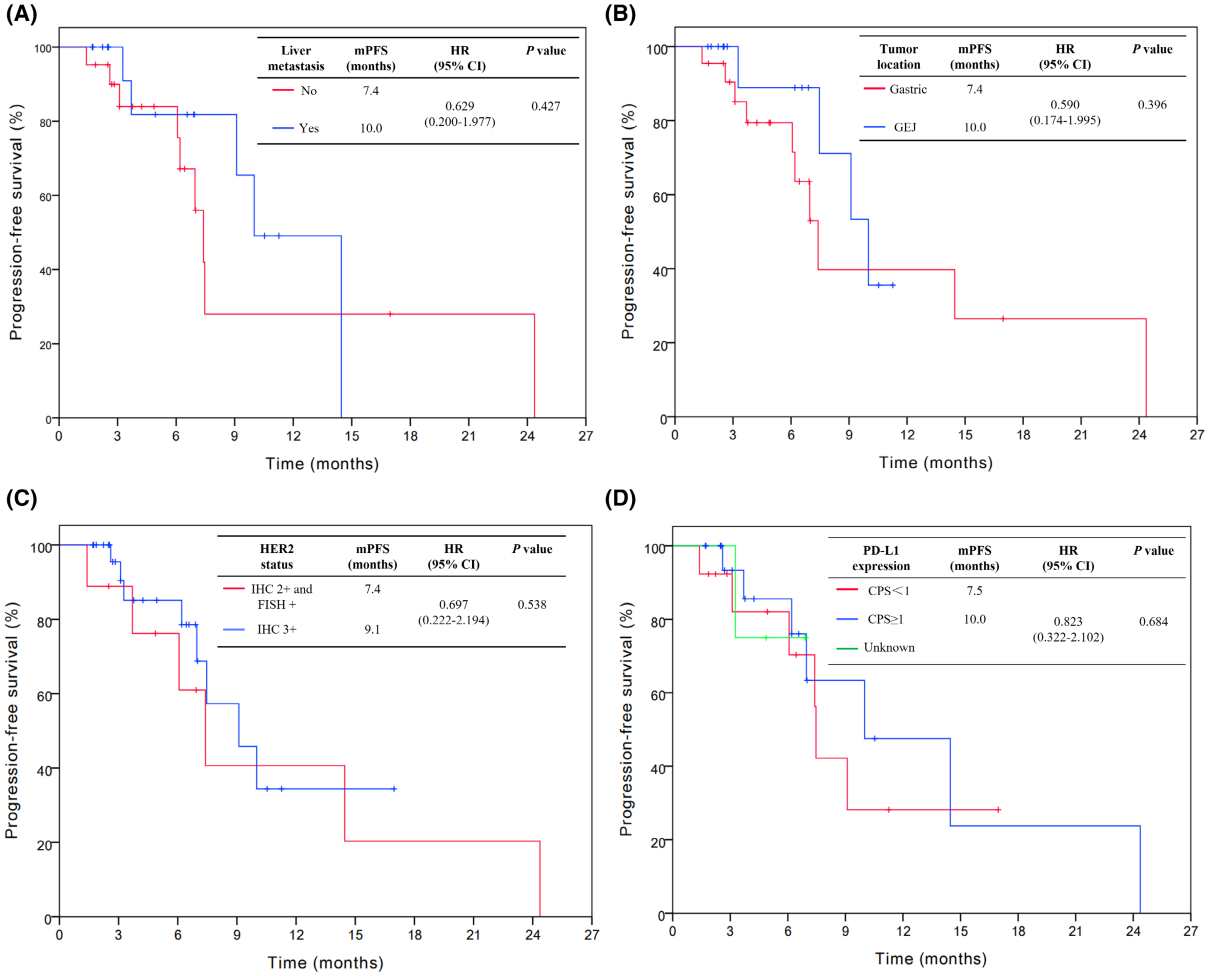
Kaplan–Meier analyses of progression‐free survival according to liver metastasis (A), primary tumor site (B), HER2 status (C), and PD‐L1 expression (D). CI, confidence interval; CPS, combined positive score; FISH, fluorescence in situ hybridization; GEJ, gastroesophageal junction; HER2, human epidermal growth factor receptor 2; HR, hazard ratio; IHC, immunohistochemistry; mPFS, median progression‐free survival; PD‐L1, programmed cell death ligand 1.

In the efficacy population, the overall mPFS was 9.1 months (95% confidence interval [CI]; 6.3–11.9 months) (Figure 3A). For the first‐line treatment, the mPFS was 10 and 7 months for the second line. The difference was not statistically significant (hazard ratio [HR] 2.126, 95% CI 0.693–6.524, *p* = 0.187) (Figure 3B). Next, we analyzed the associations between patients' and treatment‐related factors and PFS. As indicated in Figure 4A, patients with liver metastasis did show longer mPFS compared with patients without liver metastasis (10.0 vs. 7.4 months), however, the difference was not statistically remarkable (HR 0.629, 95% CI 0.200–1.977, *p* = 0.427). Similar results were shown for tumor location (GEJ vs. gastric, 10.0 vs. 7.4 months, HR 0.590, 95% CI 0.174–1.995, *p* = 0.396), HER2 status (IHC3+ vs. IHC2+ and FISH+, 9.1 vs. 7.4 months, HR 0.697, 95% CI 0.222–2.194, *p* = 0.538), and PD‐L1 expression (CPS ≥1 vs. CPS <1, 10.0 vs. 7.5 months, HR 0.823, 95% CI 0.322–2.102, *p* = 0.684) (Figure 4).

### 3.4 Treatment‐related adverse events

In general, the study therapy was well‐tolerated. There were neither grade 4 treatment‐related adverse events (TRAEs) nor treatment‐related deaths in our study. As shown in Table 3, the most common TRAE was grade 1 hypoalbuminemia (57.8%). 22.2% of patients developed grade 3 TRAEs. The most frequent grade 3 TRAEs were hypokalemia (6.7%) and thrombocytopenia (4.4%).

**TABLE 3:** Treatment‐related adverse events.

TRAEs	Grade (*N* = 45)		
	1 (%)	2 (%)	3 (%)
Hematologic			
Leukopenia	8 (17.8)	10 (22.2)	1 (2.2)
Neutropenia	7 (15.6)	8 (17.8)	1 (2.2)
Anemia	23 (51.1)	8 (17.8)	1 (2.2)
Thrombocytopenia	7 (15.6)	7 (15.6)	2 (4.4)
Lymphocytopenia	23 (51.1)	11 (24.4)	0
Non‐hematologic			
Diarrhea	2 (4.4)	2 (4.4)	0
Vomiting	0	2 (4.4)	0
Nausea	1 (2.2)	1 (2.2)	0
Decreased appetite	1 (2.2)	1 (2.2)	0
Fatigue	3 (6.7)	1 (2.2)	0
Pyrexia	1 (2.2)	0	0
Rash	4 (8.9)	1 (2.2)	1 (2.2)
Hypothyroidism	1 (2.2)	1 (2.2)	0
Hyperthyroidism	2 (4.4)	0	0
Interstitial lung disease	1 (2.2)	1 (2.2)	0
Neuropathy peripheral	0	1 (2.2)	1 (2.2)
Increased ALT	7 (15.6)	3 (6.7)	0
Increased AST	14 (31.1)	2 (4.4)	0
Hypokalemia	10 (22.2)	0	3 (6.7)
Hypophosphatemia	18 (40.0)	0	0
Hypoalbuminemia	26 (57.8)	3 (6.7)	0
Blood bilirubin increased	5 (11.1)	1 (2.2)	0

Abbreviations: ALT, alanine aminotransferase; AST, aspartate aminotransferase; TRAEs, treatment‐related adverse events.

## 4 DISCUSSION

Since the success of the phase III trial ToGA in 2010, the combination of trastuzumab and chemotherapy has been the standard therapy for advanced HER2‐positive GC.^9^ The previously reported ORR and mPFS were 41%–64% and 6.7–9.0 months, respectively.^17^, ^18^, ^19^ Our study demonstrated the high ORR (76.3%), DCR, and manageable safety for the combination of anti‐PD‐1 antibody, trastuzumab, and chemotherapy in patients with HER2‐positive gastric/GEJ cancer. To our knowledge, this is the first study to verify the role of the foresaid treatment regimen in gastric/GEJ cancer patients with HER2‐positive in a real‐world setting.

As described in the Section 3, 10 patients received the study therapy as a second‐line treatment. They developed disease progression during or within 6 months after adjuvant chemotherapy. Currently, there is no valid evidence to support the addition of trastuzumab to adjuvant chemotherapy in patients with resectable HER2‐positive GC after D2 resection. Therefore, trastuzumab is not applied to such patients in our institution. Though the study therapy was administered as a second‐line treatment, these 10 patients were immunotherapy and trastuzumab‐naïve before the study therapy. We observed a lower ORR and DCR in the second‐line treatment group, indicating the rationale for the early use of immunotherapy and trastuzumab in patients with HER2‐positive advanced GC. Data from a phase II study (T‐ACT) suggested that continuous use of trastuzumab beyond progression failed to improve PFS in patients with HER2‐positive GC.^20^ Our data indirectly supported this point. As mentioned in Section 3, the addition of anti‐PD‐1 antibody and continuous use of trastuzumab did not control disease progression after treatment failure to trastuzumab in two patients that were excluded from the efficacy population.

The high efficacy of the above study therapy is also biologically reasonable. Trastuzumab can increase the HER2 internalization and cross‐presentation by dendritic cells to promote HER2‐specific T‐cell responses.^21^, ^22^ Trastuzumab can also upregulate expressions of PD‐1/PD‐L1 and modulate the expression of major histocompatibility complex class II (MHC II).^23^ The anti‐PD‐1 antibody can combine with trastuzumab to prompt HER2‐specific T‐cell response and lead to the expansion of peripheral memory T cells.^24^, ^25^ However, a higher clinical efficacy is still challenged by some resistance mechanisms, such as heterogeneous expressions of HER2^26^ and heterogeneous distribution of trastuzumab,^27^ poor penetration of trastuzumab through blood–brain barriers,^28^ and less immunological activity in metastatic lesions in GC patients,^29^ and the immune privilege of central nervous system under normal conditions.^30^ Therefore, we wondered whether a high incidence of brain metastasis could occur in patients under the study therapy.

Patients with HER2‐positive GC tended to relapse in distant sites other than peritoneal or local recurrence.^31^, ^32^ Brain metastasis from GC is uncommon, and the incidence rate is 0.47%–0.7%.^33^, ^34^ In patients with breast cancer, there is a positive correlation between HER2 overexpression and a higher risk of brain metastasis.^35^ Previous studies showed that brain metastases were higher and relapsed more frequently in HER2‐positive GC patients.^36^, ^37^ The brain metastasis was diagnosed in three patients by brain MRI or cerebrospinal fluid examination after the appearance of nervous system disorders. We observed a relatively high incidence of brain metastasis (21.4%, 3/14) in patients who failed the study therapy. Oncologists should carefully differentiate between brain metastasis and chemotherapy‐induced refractory nausea and vomiting. Once nervous system disorders appear, intensified follow‐up and brain MRI should be considered to ensure an early diagnosis of brain metastasis.

Though impressive responses were achieved in some patients, there were still some patients not sensitive to the study therapy or developing resistance after response, indicating the need to identify predictive biomarkers for the patients' outcomes. Both the Panthera trial and another phase II trial tried to find some biomarkers for the study therapy. These two clinical trials indicated that there was no obvious correlation between PFS and the PD‐L1 expression.^10^, ^38^ And similar results were also found in our study. Given the high efficacy of the study therapy, we infer that the use of immunotherapy should be considered in HER2‐positive GC patients regardless of the PD‐L1 expression level.

In our institution, the HER2‐positive rate was 11.4% in patients with GC, which seemed less than the data reported by the Phase III trial ToGA^9^ but was similar to other Chinese patients based studies.^6^, ^7^ We thought the actual HER2‐positive rate would be slightly higher than 11.4% because 72 patients in our study were with HER2 IHC 2+ but were absent from the FISH test. Most of them only received adjuvant chemotherapy in our institution and chose to skip the FISH test, which was not covered by insurance.

Our study had some limitations. First, although we retrospectively collected over 1000 gastric/GEJ cancer patients in our institution during the past 3 years, only about 50 patients were recruited for analyses, which was due to the lower HER2‐positive rate in all gastric/GEJ cancer patients. Second, similar to other retrospective studies, it was hard to collect all the adverse events information due to the incomplete records during treatment. In addition, this was a single‐arm study without a control cohort. NGS was not performed to validate the potential biomarkers. Potential mechanisms were not included in our study. We are currently investigating the effects of the study therapy on reprogramming the tumor immune microenvironment, and related data will be released shortly.

In summary, our study manifested the high effectiveness of the combination regimen with anti‐PD‐1 antibody, trastuzumab, and chemotherapy in patients with HER2‐positive GC in a real‐world setting for the first time. In patients who failed this regimen, an increased incidence of brain metastasis was observed.

## AUTHOR CONTRIBUTIONS

**Ju Yang:** Conceptualization (equal); data curation (equal); visualization (lead); writing – original draft (lead); writing – review and editing (lead). **Zhan Shi:** Data curation (lead); investigation (equal); visualization (equal); writing – original draft (equal). **Xin Zhang:** Data curation (equal). **Qin Liu:** Data curation (equal). **Xiaobin Cui:** Formal analysis (equal). **Lin Li:** Formal analysis (equal). **Baorui Liu:** Conceptualization (equal); funding acquisition (equal); project administration (equal); supervision (equal). **Jia Wei:** Conceptualization (equal); funding acquisition (equal); project administration (equal); writing – review and editing (lead).

## FUNDING INFORMATION

This work was funded by grants from the National Natural Science Foundation of China (82073382), Fund for Distinguished Young Scholars of Jiangsu Province (BK20190001), Program of Jiangsu Provincial Key Medical Center (YXZXB2016002), and the Fundamental Research Funds for the Central Universities (0214‐14380506).

## CONFLICT OF INTEREST STATEMENT

The authors declare that they have no conflict of interest.

## ETHICS STATEMENT

All procedures performed in this study involving human participants were in accordance with the guidelines of the Ethics Committee of Nanjing Drum Tower Hospital and with the 1964 Helsinki Declaration.

## CONSENT FOR PUBLICATION

We confirm that the manuscript has been reviewed and approved by all named authors for publication.

## Data Availability

The data that support the findings of this study are available from the corresponding author upon reasonable request.
